# Active Smarter Kids (ASK): Rationale and design of a cluster-randomized controlled trial investigating the effects of daily physical activity on children’s academic performance and risk factors for non-communicable diseases

**DOI:** 10.1186/s12889-015-2049-y

**Published:** 2015-07-28

**Authors:** Geir K Resaland, Vegard Fusche Moe, Eivind Aadland, Jostein Steene-Johannessen, Øyvind Glosvik, John R Andersen, Olav M Kvalheim, Heather A McKay, Sigmund A Anderssen, on behalf of the ASK study group

**Affiliations:** Faculty of Teacher Education and Sports, Sogn og Fjordane University College, Sogndal, Norway; Faculty of Health Studies, Sogn og Fjordane University College, Førde, Norway; Centre of Health Research, Førde Central Hospital, Førde, Norway; Department of Chemistry, University of Bergen, Bergen, Norway; Department of Family Practice, Faculty of Medicine, The University of British Columbia, Vancouver, Canada; Department of Sports Medicine, Norwegian School of Sport Sciences, Oslo, Norway

**Keywords:** Cluster-randomized trial, Children, Academic performance, Cognitive functions, Executive functions, Non-communicable diseases, Physical activity, Sedentary behavior, Cardiorespiratory fitness, Quality of life

## Abstract

**Background:**

Evidence is emerging from school-based studies that physical activity might favorably affect children’s academic performance. However, there is a need for high-quality studies to support this. Therefore, the main objective of the Active Smarter Kids (ASK) study is to investigate the effect of daily physical activity on children’s academic performance. Because of the complexity of the relation between physical activity and academic performance it is important to identify mediating and moderating variables such as cognitive function, fitness, adiposity, motor skills and quality of life (QoL). Further, there are global concerns regarding the high prevalence of lifestyle-related non-communicable diseases (NCDs). The best *means to address this challenge could be through primary prevention.* Physical activity is known to play a key role in preventing a host of NCDs. Therefore, we investigated as a secondary objective the effect of the intervention on risk factors related to *NCDs. The purpose of this paper is to describe the design of the ASK study, the ASK intervention as well as the scope and details of the methods we adopted to evaluate the effect of the ASK intervention on 5*^*th*^*grade children.*

**Methods & design:**

The ASK study is a cluster randomized controlled trial that includes 1145 fifth graders (aged 10 years) from 57 schools (28 intervention schools; 29 control schools) in Sogn and Fjordane County, Norway. This represents 95.3 % of total possible recruitment. Children in all 57 participating schools took part in a curriculum-prescribed physical activity intervention (90 min/week of physical education (PE) and 45 min/week physical activity, in total; 135 min/week). In addition, children from intervention schools also participated in the ASK intervention model (165 min/week), i.e. a total of 300 min/week of physical activity/PE. The ASK study was implemented over 7 months, from November 2014 to June 2015. We assessed academic performance in reading, numeracy and English using Norwegian National tests delivered by The Norwegian Directorate for Education and Training. We assessed physical activity objectively at baseline, midpoint and at the end of the intervention. All other variables were measured at baseline and post-intervention. In addition, we used qualitative methodologies to obtain an in-depth understanding of children’s embodied experiences and pedagogical processes taking place during the intervention.

**Discussion:**

*If* successful, ASK could provide strong evidence of a relation between physical activity and academic performance that could potentially inform the process of learning in elementary schools. Schools might also be identified as effective settings for large scale public health initiatives for the prevention of NCDs.

**Trial registration:**

Clinicaltrials.gov ID nr: NCT02132494. Date of registration, 6^th^ of May, 2014.

## Background and objectives

The relation between physical activity and academic performance has received widespread attention owing to the increasing pressure on schools and teachers to provide children with a range of physical and intellectual capabilities. Physical activity might affect children’s academic performance [1] and cognitive function [2]. The school setting is the cornerstone of societies globally. Schools reach a diverse group of children from an early age, and provide a learning environment that can exert an influence on children across a long period of time. Therefore, schools provide potentially useful settings to implement strategies designed to increase children’s physical activity [3, 4].

Over the last decade, there has been a considerable increase in both the number and quality of school-based studies. However, as school based interventions are difficult to implement, many have not assessed academic performance or cognition with validated tests, others have lacked a theoretical framework or were not randomized, some were of short duration, delivered a small “dose” of physical activity, had a small sample size or involved promotion of physical activity by non-experts. Also, it should be noted that relatively few studies have measured physical activity objectively [1]. Hence, there is a need for high-quality studies that address these limitations.

Further, there are global concerns regarding the high prevalence of lifestyle-related non-communicable diseases (NCDs) [5]. The prevalence of *NCDs,* such as type 2 diabetes, is increasing worldwide, and they affect people of all ages, including children [6]. The ultimate costs are a decreasing quality of life and escalating healthcare expenditures [7]. *P*hysical activity is known to effectively prevent a host of NCDs [8], thus providing an important target for primary prevention.

Our primary objective is to investigate the effect of a one year school-based physical activity intervention (Active Smarter Kids; ASK) on academic performance on a sample of 10-year-old boys and girls attending elementary school in Norway. Due to the complex relation between physical activity and academic performance, we identified possible mediating and moderating variables (cognitive function, physical fitness, adiposity, motor skills and quality of life (QoL)).

Our secondary objective is to investigate the effect of ASK on modifiable lifestyle-related risk factors related to *NCDs, such as* physical activity and sedentary behavior, cardiorespiratory fitness, muscle strength, motor skills, adiposity, dyslipidemia and blood pressure.

## Methods/Design

### A. Study design, study population and inclusion criteria

The ASK study is a seven months cluster-randomized parallel group controlled trial, with random allocation at the school level with a 1:1 ratio. All children are in fifth-grade (10-year-olds) from the Sogn and Fjordane County, situated in the *western* part of Norway. Inclusion criteria were that schools should have at least seven children in fifth-grade; that children were healthy (with no serious or chronic illnesses) and able to participate in daily physical activity and physical education (PE). Participants had to be able to complete standard academic performance tests (our primary outcome).

Sixty schools, encompassing 1202 fifth-grade children, fulfilled the inclusion criteria, and agreed to participate. This represented 86.2 % of the population of 10-year-olds in the county, and 95.2 % of total possible recruitment. We randomized 30 schools for the intervention (I-schools) and 30 schools for the control (C-schools) arm. A neutral third party (Centre for Clinical Research, Haukeland University Hospital, Norway) performed the randomization. After randomization, three schools (two I-schools and one C-school) from the same municipality declined to participate. In total, 1145 (97.4 %) of 1175 children from 57 schools (28 I-schools and 29 C-schools) agreed to participate in the study.

### B. The ASK intervention (dose, intensity, teacher training and following up)

#### Dose

The ASK intervention consists of three components (in total 165 min/week). In order to optimize adherence, these were established as part of the mandatory school curriculum for all children attending I-schools:

**1)** ASK physically active educational lessons (3 × 30 min each week); Academic lessons in three core subjects, Norwegian, *mathematics* and English carried out in the school playground. **2)** ASK physical activity breaks during classroom lessons (5 min × 5 days each week). **3**) ASK physical activity homework prepared by the teachers (10 min daily; 5 × 10 min each week).

As a part of the mandatory school curriculum in Norway, children from both the I-schools and C-schools participated in curriculum-prescribed 90 min/week of PE and 45 min/week of physical activity, in total; 135 min/week. Therefore, the children from the I-schools performed 300 min/week of physical activity/PE, while the children from the C-schools performed 135 min/week of physical activity/PE. However, it was specified to the C-school that they could carry out the amount of physical activity/PE that they would have done regardless of the ASK study.

#### Intensity

The three physical activity components in the ASK intervention were planned so that activities were varied and enjoyable for the children. We emphasized to the ASK-teachers that all activities should include all children, especially those who were not particularly fit or enthusiastic about physical activity. Special attention was given to creating an encouraging and motivating atmosphere during lessons, in order to support positive feelings and attitudes towards physical activity. Approximately 25 % of daily physical activity in the ASK intervention was intended to be of vigorous intensity. This was defined as “children would be sweating and out of breath”. The vigorous activity component was achieved by selecting a variety of high intensity activities such as running, relay racing, obstacle courses and various forms of active play.

Fifty-nine ASK teachers led the physical activity component in the 28 I-schools. These ASK teachers are classroom teachers assigned by the school principal to teach 5th grade in the I-schools (independently of the ASK study). To ensure that teachers were empowered, supported and qualified to deliver the ASK physical activity intervention to their students, we conducted three comprehensive instructional seminars (April, June and September 2014) for the I-schools teachers. Further, we provided two regional refresher sessions during the intervention period (December 2014 and February 2015) to encourage teachers to share experiences and solve challenges together with each other and the research team. Finally, we provided teachers in I-schools with email- and telephone-support. We also provided a password protected ASK homepage (http://www.askstudy.no) that supplied teachers in I-schools with information, videos and physical activity lessons.

### C. Theoretical framework

The ASK study is embedded in a socio-ecological conceptual framework that focuses on positive physical activity behaviors [9]. In brief, the socio-ecological model acknowledges the role of proximal (e.g. individual and social) and more distal (e.g. school physical environment, school policy) determinants of health behavior change as necessary to achieve sustained positive health behaviors. To address individual and social determinants we adopted specific theoretical frameworks including Harter’s Competence Motivation Theory [10], Achievement goal theory [11] and Ryan & Deci’s self-determination theory [12]. In line with these theories the ASK intervention emphasizes creating autonomy supporting and mastery oriented teacher-student interaction in order to enhance students’ physical activity behavior by positively influencing their perception of competence, self-efficacy, and intrinsic motivation for physical activity.

### D. Outcome measures

All participating children were tested at baseline and post intervention. The exception was physical activity which was assessed at baseline, at the midpoint of the intervention period and at post intervention. We describe each variable assessed in the following sections.

### Academic performance (primary outcome)

Academic performance in **1)** reading, **2)** numeracy, and **3)** English was measured using specific standardized Norwegian National tests designed and administrated by The Norwegian Directorate for Education and Training (NDET) [13]. The three different tests were administered on three different days, both at the baseline and post intervention test. Tests are extensively verified for validity and reliability by NDET and are aligned with competencies demanded from all schools by the national curriculum. For ASK, we analyzed reading, numeracy and English individually and as a composite score.

### Cognitive function/Executive functions

We assessed the three core executive functions identified by Miyake et al. (2000) [14], i.e. inhibition, cognitive flexibility and working memory using four pen and paper tests. The tests were administered in a quiet room at the children’s school. All tests required 15–20 min to complete. **1)** To assess inhibition we used Golden’s version of the Stroop test [15, 16]. **2)** To assess cognitive flexibility we used two tests, one verbal (Verbal fluency) and one nonverbal test (The Trail Making Test) [17]. **3)** To assess working memory we used a digit span test with digits both forward and backward (Wechsler Intelligence Scale for Children, 4th ed; WISC-IV) [16].

### Physical activity and sedentary behavior

Physical activity was measured by triaxial accelerometry (ActiGraph GT3X+, LLC, Pensacola, Florida, USA). ActiGraph accelerometry uses the most widely applied instrument for objective assessment of physical activity and has been extensively tested for validity and reliability in children [18]. Children were instructed to wear the accelerometer on the right hip over seven consecutive days at all times, except during water activities or while sleeping. A wear-time of ≥ 480 min/day was applied as a criterion for a valid day. Periods of ≥ 20 min of zero counts are defined as non-wear time [19]. The number of ‘valid days’ vary depending on the analyses performed. Outcomes for physical activity levels are total physical activity level (counts/min), sedentary behavior (min/day and percentage of valid wear time), light physical activity, moderate physical activity, moderate to vigorous physical activity and vigorous physical activity (min/day), using previously applied and established cut points [20, 21]. All analyses were based on accumulation of data over 10 s epochs.

### Cardiorespiratory fitness

Cardiorespiratory fitness was measured with an intermittent practicable running field test (the Andersen-test [22]) that has demonstrated reliability and validity for our target age group [23]. The Andersen-test was administered as per standard procedures. The children were tested indoors on a wooden or rubber floor in groups of 10–20 children. The test required 10 min to perform. Children ran from one end line to another (20 m apart) in an intermittent to-and-fro movement, with 15-s work periods and 15-s breaks (standing still). We recorded the distance covered as the outcome for analysis.

### Muscle strength

Muscle strength (i.e., endurance, isometric and explosive strength) was measured using reliable and validated selected tests from the Eurofit test battery [24]: **1)** Upper limb strength – handgrip strength using a hand dynamometer (Baseline® Hydraulic Hand Dynamometer, Elmsford, NY, USA); 2**)** Explosive strength in the lower body, standing broad jump. Muscle strength tests were recorded both individually and as a composite score for analyses.

### Motor skills

Motor skills were measured using a battery of three tests: **1)** Catching with one hand and throwing at a wall target constitute the aiming and catching subgroup of the movement-ABC-2; **2)** Throwing at a wall target; **3)** Ten times 5 m sprint. Tests **1)** and **2)** are from the Movement Assessment Battery for Children 2 (Movement ABC-2), age band 3 (11–16 years) [25], and test **3)** is from Eurofit test battery [24]. Reliability and validity of these tests are well documented [26–29]. The Movement ABC-2 demonstrated good concurrent and convergent validity [30, 31]. We recorded motor skills tests both individually and as a composite score for analyses.

### Anthropometry and maturity

Body mass (weight; 0.1 kg) was measured using an electronic scale (Seca 899, SECA GmbH, Hamburg, Germany) with children wearing light clothing. Stature (height; 0.1 cm) was measured with a portable Seca 217 (SECA GmbH, Hamburg, Germany). The child was asked to face forward, with shoes removed. We calculated body mass index (kg · m^−2^) as weight (kg) divided by the height squared (m^2^).

Waist circumference was measured with a Seca 201 (SECA GmbH, Hamburg, Germany) ergonomic circumference measuring tape. The measure was taken two cm over the level of the umbilicus (0.5 cm) with the child’s abdomen relaxed at the end of a gentle expiration. The child stood with arms hanging slightly away from the body. We collected two measurements from each child. If the difference between measures was a greater than one cm, we obtained a third measurement; the mean of two closest measurements are used for analyses.

Body fat was measured using four skinfold thickness sites (biceps, triceps, subscapular, and suprailiac) on the left side of the body using a Harpenden skinfold caliper (Bull; British Indicators Ltd., West Sussex, England) as per the criteria described by Lohmann et al. [32]. The Harpenden skinfold caliper has been tested for validity and reliability in children [33]. All measurements were conducted in a private room. The caliper was placed around the skinfold one cm below and to the left where the skin was held between thumb and forefinger. We collected two measurements at each site (in sequence). If the difference between measures was a greater than two mm, we obtained a third measurement; the mean of the two closest measurements are used for analyses. The skinfold calipers was calibrated each time they were moved between measurement sites.

Children self-assessed their pubertal stage as per the Tanner method [34] using a scale of color images proposed by Carel and Leger [35]. Children were given standardized series of images with explanatory text. We used a scale based on pictures of pubic hair for both sexes and of breast and genital development for girls and boys, respectively. We created a relaxed atmosphere for this assessment and the researcher was of the same sex as the child. In a private room, children were asked to read the brief descriptions for each stage, and instructed to put a checkmark in the box below the picture that best represented their stage of development for each component.

### Blood pressure

Systolic and diastolic blood pressure (BP) was measured using the Omron HBP-1300 automated BP monitor (Omron Healthcare, Inc, Vernon Hills, IL, US). The device is validated as per the AAMI protocol using the ANSI/AAMI/ISO standard (Omron, 2015) [36]. Children rested quietly for ten minutes in a sitting position with no distractions before BP was measured. During measurement, each child sat in a quiet room; BP was measured on the upper right arm using an appropriate sized cuff. We took four measurements, with a one-minute interval. We used the mean of the last three measurements for analyses. If a difference > five mmHg between measurements was found, we obtained one extra measurement, in which case the mean of the last four are used.

### Blood samples (factors impacting risk of metabolic disease)

After an overnight fast, between 08:00 a.m. and 10:00 a.m., a nurse or phlebotomist collected an intravenous blood sample from each child’s antecubital vein. Serum was obtained according to a standardized protocol consisting of the following steps: 1) Blood plasma was collected in 5 ml tubes with gel (Vakuette® Serum Gel with activator, G456073). 2) Tubes were carefully turned upside-down five times and placed vertically for coagulation. 3) After 30 minutes the sample was centrifuged at 2000 G for 10 minutes. Serum was then visually inspected for residues and centrifugation was repeated if residue was present. 4) The serum tube was kept in refrigerator at 4 °C before pipetting 0,5 ml into cryo tubes. 5) The cryo tubes were then stored at -80 °C prior to biochemistry analyses. 

Serum samples were analyzed for constituents related to traditional risk factors for NCDs, such as insulin, glucose and the standard lipid panel (triglycerides, total cholesterol, high density lipoprotein cholesterol (HDL) and low density lipoprotein cholesterol (LDL)) using standard laboratory methods. Baseline and post intervention samples were analyzed at the same time in one batch at an ISO certificated laboratory.

Analyses of low molecular weight metabolites and lipoprotein distribution was performed for serum samples using proton nuclear magnetic resonance (1H-NMR) spectroscopy. The proton NMR profiles of low molecular metabolites (fatty acids and amino acids) were obtained by using a standard experimental set-up [37]. For lipoproteins we made a minor modification to the experimental protocol used by Mihaleva et. a.l (2014) [38]. Lipoprotein features such as subclass concentrations for HDL, LDL etc. and average particle size of each main class were derived from the NMR profiles. Fatty acid analysis was performed according to the following protocol: 200 µL serum sample is weighed into 10 mL glass tubes and water evaporated under nitrogen, followed by adding 150 µL internal standard (triheptadecanoin, 0.4855 mg/mL). After evaporating the solvent the samples are derivatized to fatty acid methyl esters (FAME) by direct esterification in methanolic HCl at 90 °C for 2 h under nitrogen atmosphere [39]. FAMEs will be extracted and analyzed by gas chromatography as described in Gudbrandsen et al. [40]. The samples are run in a randomized sequence and the FAME reference mixture GLC-461 (Nu-Chek Prep, Elysian, MN, USA) will be analyzed as every 10th sample. Chromatographic areas are corrected by empirical response factors calculated from the GLC-461 mixture. The amounts of FAs are thereafter quantified by means of the internal standard. The total amounts of FAs in each serum sample are converted to amounts in μg per g sample by dividing with the sample weights. 

### Questionnaires: children, parents/guardians, teachers

#### Children

Mode of transport to school, levels of leisure time physical activity, sedentary behavior and psycho-social and environmental correlates of physical activity were assessed with a questionnaire developed for youths also used in a large national representative surveillance study of physical activity among Norwegian children and youth [41, 42].

Children’s QoL was assessed by self-report using the Kidscreen-27 questionnaire [43], which consists of 27 items covering the following five QoL dimensions: **1**) physical well-being (5 items); **2**) psychological well-being (7 items); **3**) parents/guardians relations & autonomy (7 items) **4**) social support & peers (4 items); and **5**) school environment (5 items). The Kidscreen-27 questionnaire was developed simultaneously in several European countries, and has been validated in Norwegian children aged 10 [44] and 8–18 [45] In order to assess well-being at school and predictors for this, we also used the teacher and classmate support scale [46].

Physical activity preferences were assessed with a questionnaire created by the ASK group. The children rated their preferences for each of 28 different activities on a scale from one to ten.

#### Parents/guardians

We obtained self-reported height and weight from parents/guardians. We assessed physical activity habits, physical activity in leisure time and everyday living with a questionnaire developed for adults [42]. This questionnaire also assessed background variables (ethnic background, income, education and job description), as well as psychological, social and environmental determinants of physical activity.

#### Teachers

To assess behavioral self-regulation of children from I-schools, we used 10 items from the Child Behavior Rating Scale (CBRS) [47]. The original CBRS was designed to evaluate a child’s task behavior and social behavior with peers and adults [48]. More recent studies have identified a 10-item factor that describes child behavioral self-regulation in a classroom setting [49]. Teachers were asked to rate children’s practical behavior on a five point Likert scale ranging from 1 (never) to 5 (always) to indicate how frequently a given behavior occurs. The CBRS is a reliable and valid tool that has been used in multiple studies in Western countries [50, 51]. After the intervention period, the teachers from the I-schools were asked to provide a self-report survey evaluation of the ASK intervention.

### Qualitative study of embodied experiences and pedagogical processes

We investigated the embodied experiences and interpersonal relationships of teaching and learning in physical activity from the perspectives of children, teachers and parents/guardians using qualitative methodologies. The purpose was to obtain an in-depth understanding of pedagogical processes taking place in the ASK study. More specifically, we identified purposive sample of classes from two I-schools and two C-schools. The sample criteria emphasized variation in school location, school and class size, teachers’ education and experience with PE and physical activity, and the schools’ overall focus on physical activity. In total 100 children with parents’/guardians’ approval and seven teachers provided informed written consent to participate. All children participated in a drawing and writing task and one group interview. The students were also observed by the researcher during one PE lesson. This data, together with teachers’ description of their students in PE lessons, were used to choose children to further in-depth studies.

We conducted in-depth interviews and field observations including short video recordings with 32 children. Teachers were interviewed (individually and in groups) and observed; we also interviewed two groups of parents. We deem insight attained using multiple methods and perspectives to be essential in research with children, as adult researchers may have difficulty interpreting a child’s perspective [52, 53]. Further, a deeper understanding of childrens’, teachers’, and parents’/guardians’ perspectives enriched and gave a nuanced picture of processes leading to certain outcomes from the main trail. Data (i.e., transcripts of interviews, field notes, drawings and writings, pictures and video recordings) were structured and explored using the software program Nvivo 10 (QSR International Pty Ltd 2015). Developing in-depth knowledge is an iterative process where preliminary interpretations of the empirical material are brought together with discussions on theoretical significance in increasing detail.

We consider empirical data as a construct created in a specific cultural and historical context, influenced by the interplay between the researcher and the participant, and the researcher’s theoretical underpinning. Empirical data are used as “a critical dialogue partner” and as an inspiration to challenge and rethink established taken-for-granted assumptions and theories [54]. Thus our approach differed from a more conventional approach where empirical data are seen as separate from theory, but are used to guide and validate theory development. By these means we aim to interpret the children’s embodied experiences and pedagogical practices during the ASK intervention as an interactive whole where researchers’ pre-understanding, alternative theoretical input, and empirical data constitute the dialogue setting [54].

We further created a reflexive account on children’s embodied experiences and pedagogical practices during the ASK intervention. Thus, the qualitative approach thus shed light on «what’s going on» in schools during the intervention period: How do children interact with each other and the teachers within the schools during the intervention, and, in particular, how is it to be a child in an I-school? In addition, this gives us a basis to discuss the teaching and learning processes, the possible benefits and pitfalls in the intervention, as well as to evaluate whether the intervention and the ASK study as a whole might serve as tools towards creating an improved atmosphere for learning in the schools in the future.

### E. Adherence

Regarding adherence to the intervention program, school administrators at the 57 schools recorded every child’s absence from school. Administrators also recorded children’s injuries and the names of children who did not participate in physical activity lessons.

We assessed the extent to which the intervention was implemented (dose), the quality of the implementation (fidelity), as well as feasibility as perceived by teachers and others.

ASK teachers at the 28 I-schools completed a report each week that described activities performed throughout the school day, the intensity of the activities (on a 1 to 3 scale) and the number of minutes allocated to physical activity/PE in each ASK session. All 29 C-schools, at the end of the school year, completed a report that describes the activities that were performed and the estimated time allocated to physical activity/PE during the school year (minutes/week).

### F. Statistical considerations/Statistical methods

#### Randomization procedure

The randomization procedure is explained in section 2a.

#### Blinding

Blinding of children and schools was not possible due to the nature of the experiment. However, only the project management group has formal knowledge of group assignment. The data manager and statisticians are blinded to group allocation until analyses are conducted.

#### Power calculations/Sample size and power

The ASK study was designed to detect an effect size (Cohen’s D) of 0.35 between two groups for change in academic performance. Sample size calculations were performed using standard formulas, given ɑ = 0.05, 1-β = 90 %, group ratio 1:1 and correlation of repeated measurements = 0.7, leading to a naive n = 103 children in each group. Due to the cluster-randomized controlled trial (RCT) design, the sample size was corrected for the design effect using *formula 1;* Design Effect = 1 + [(CV^2^ + 1)*n – 1]* (ICC), where CV = coefficient of variation for n, n = number of children within each cluster, and ICC = intraclass correlation coefficient. Thus, after accounting for school clusters, we required a sample size of 468 children in each arm (INT; CON). This is based on an ICC = 0.15, n = 16.2 after accounting for 20 % attrition at the subject level, and CV = 0.72 (design effect correction factor = 4.54). The assumed ICC is a conservative estimate compared to the expected ICC of 0.03 to 0.07, which lay the foundation for the sample size of the A+ PAAC study [55]. For secondary outcomes we assume an ICC of 0.05 to 0.10 [56]. Accepting the assumptions above, the target sample size allowed us to detect a significant difference between the groups for variables reaching an effect size of 0.24 (ICC = 0.05) to 0.30 (ICC = 0.10) or higher.

#### Plan for analysis

Our main analysis in order to assess the effectiveness of the ASK intervention are based on and intention to treat analysis [57, 58]. We also acknowledge the importance of conducting per-protocol analyses to determine efficacy based on schools that showed fidelity to the model and performed the physical activity intervention as intended [59]. Therefore, we conducted secondary analyses of children who participated in ≥80 % of the physical activity-based intervention and schools which delivered ≥80 % of the physical activity-based intervention as we deem this acceptable adherence to the intervention itself.

We describe missing data using appropriate flow charts [60] that also allowed for investigation of missing data mechanisms and assumptions that underpin statistical analyses. Missing data was imputed from all available relevant variables by means of multiple imputation using a Markov Chain Monte Carlo procedure with 20 iterations, with an assumption that data are missing at random. We performed sensitivity analyses to evaluate the stability of results under various assumptions of missing data.

We used a mixed-effects model to test the between-group difference (intervention vs. control) for the main outcome (academic performance), controlling for baseline values and including school as a random effect. The per-protocol analysis was adjusted for differences between groups as appropriate.

We conducted our analyses using the newest available version of IBM SPSS (IBM SPSS Statistics for Windows, Armonk, NY: IBM Corp., USA). A two-sided p-value ≤ 0.05 were considered statistically significant.

We conducted defined moderator-mediator analyses a priori to evaluate characteristics of children and pathways that could influence the effect of the intervention on academic performance. We identified potential moderators that include both characteristics of the children and baseline values for specific secondary outcomes. We included change in executive function, motor skills, physical activity, physical fitness, adiposity and other risk factors for lifestyle-related non-communicable diseases in mediation-analyses. We used a similar strategy to evaluate moderators and mediators of secondary outcomes. As a part of the investigation of possible moderators of the effects evaluated, we conducted subgroup-analyses defining subgroups by quintiles or quintile split and testing group*subgroup interaction terms. For mediation analyses we used structural equation modeling. We also used multivariate linear regression and exploratory analyses [61, 62] to evaluate associations between secondary outcomes and academic performance, and to explore associations among independent variables.

### G. Recruitment and anchoring

Implementation research suggests that to successfully implement a large scale study within the school system, researchers must engage partners early and stakeholders should be active participants in design, implementation and dissemination of the ‘innovation’ [63]. Importantly, school-based models will be more likely to be sustained if they are anchored and supported by policy within the school system [64]. We carried out a four-stage plan to achieve this.

First, the study was accepted by “*The forum for development in kindergartens and schools in Sogn and Fjordane County”.* This educational forum was established in 1997 in Sogn and Fjordane County as an arena to discuss, translate and integrate educational policies regarding school quality and school development. The forum includes professional executives of the most important educational authorities and organizations in the Sogn and Fjordane, and it is highly respected by school administrators and teachers in the county. Second, through the “*The forum for development in kindergartens and schools in Sogn and Fjordane County”* we obtained access to a series of already established meeting venues for all county school leaders and principals, who accepted the study at the operations level. Third, parents/guardians were invited to meet with us at individual schools, where we provided a description of the study (oral and written), including our aims and any possible hazards, discomfort, or inconvenience. We also clearly described the measurement protocol and procedures and addressed any questions. We emphasized that children were free to withdraw from the study or from any measurements at any time, without providing an explanation. Fourth, we carried out an extensive anchoring process with the ASK teachers in the I-schools.

### H. Ethical perspectives

Procedures and methods used in the ASK study conform to the ethical guidelines defined by the World Medical Association’s Declaration of Helsinki and its subsequent revisions [65]. The study protocol was approved by The Regional Committee for Medical Research Ethics. The ASK study adopted a population-based approach that focuses on- and treats all children equally and considerately. We obtained written consent from each child’s parent/guardian prior to all testing. Reporting are anonymous and it is not be possible to identify individual participants in any published materials.

## Discussion

The school setting offers a unique opportunity for structured physical activity for children; it may well be the ideal environment for population-based physical activity interventions. Most children and adolescents, aged six to 16, spend most of their day in school. No other institution has possibly as much influence on children during the first two decades of their lives [66]. Further, schools might be the only means in diverse and pluralistic societies to reach a large number of children from all socio-economic backgrounds, irrespective of their parents/guardians’ behavior and attitude towards physical activity. As the physical activity is mandatory in the 28 I-schools in the ASK study, all children were involved in a physical activity intervention, and not only the motivated children. Additionally, the school offers a safe environment and facilities in an arena designed for learning. This creates an optimal context to support increased physical activity levels of children.

A comprehensively designed and appropriately powered RCT of elementary school-based physical activity with a long enough intervention and an adequate dose delivered by trained teachers in order to enhance physical activity of students would provide Level I evidence to support the effectiveness of such models. High recruitment levels and carefully selected and implemented measures (such as objectively measured physical activity) also enhance the quality of the ASK study. The trial is important for a number of reasons and may have an impact in a number of ways. First, if the physical activity intervention positively influences academic performance, this could in turn influence how the school community designs and implements children’s learning processes and related school curriculum and policies. Second, schools could be considered key sites for preventive public health initiatives that are adaptable, feasible and embedded within school culture. Academic performance and public health initiatives are interrelated. It is clear that if schools are to play a role in the prevention of NCDs and other public health problems in the future, the approach to achieving this must be anchored and accepted among the larger school community (parents, teachers, administrators). For schools to adopt this role and redefine themselves, the pathway to this is likely through enhanced academic performance through physical activity.

Importantly, if classroom teachers receive comprehensive education beyond qualification, the ASK model can be inexpensively disseminated to schools. In national and international contexts, the ASK study has the potential to extend current evidence and inform the political and scientific debate as to whether embedding physical activity into school culture is an effective next step toward meeting both educational and health goals.

## Abbreviations

ASK: Active smarter kids
BMI: Body mass index
Cm: Centimeter
CBRS: Child behavior rating scale
CV: Coefficient of variation
HDL: High density lipoprotein cholesterol
ICC: Intraclass correlation coefficient
kg: Kilogram
LDL: Low density lipoprotein cholesterol
Movement ABC-2: Movement Assessment battery for children 2
NCDs: Non-communicable diseases
PE: Physical education
QoL: Quality of live
RCT: Randomized controlled trial
I-schools: The intervention group
C-schools: The control group
